# Smarce1 fine-tunes cardiomyocyte proliferation in the embryonic zebrafish heart

**DOI:** 10.3389/fcell.2025.1636944

**Published:** 2025-08-29

**Authors:** Deung-Dae Park, Tillman Dahme, Leonie Krieg, Steffen Just, Wolfgang Rottbauer

**Affiliations:** ^1^ Molecular Cardiology, Department of Internal Medicine II, University of Ulm, Ulm, Germany; ^2^ Department of Internal Medicine II, University of Ulm, Ulm, Germany

**Keywords:** cardiomyocyte, proliferation, *smarce1*, switch/sucrose non-fermentable complex, zebrafish, cardiac development, hyperplasia

## Abstract

**Introduction:**

The molecular mechanisms regulating cardiomyocyte (CM) proliferation during heart development are essential for understanding regenerative processes but remain incompletely defined. While adult mammalian CMs are post-mitotic, zebrafish retain proliferative capacity throughout life. We aimed to identify genetic regulators that fine-tune CM proliferation during cardiac development.

**Methods:**

Using an N-ethyl-N-nitrosourea (ENU) mutagenesis screen in zebrafish, we identified the embryonic-lethal mutant *heart of stone (hos)*, which exhibits cardiac hyperplasia. Genetic mapping revealed a point mutation in *smarce1*, a component of the SWI/SNF chromatin remodeling complex. We performed morpholino knockdown, mRNA rescue, and Tet-On-driven myocardium-specific overexpression, alongside immunofluorescence, EdU labeling, qPCR, and Western blot analyses.

**Results:**

Loss of *smarce1* function in hos mutants and morphants induced ventricular CM hyperproliferation without hypertrophy. Conversely, overexpression of *smarce1*—both globally and in a myocardium-specific, inducible manner—reduced CM proliferation. Exogenous *smarce1* mRNA injection rescued the hyperproliferative phenotype in hos mutants, normalizing CM numbers and mitotic index.

**Discussion:**

These findings identify *smarce1* as a cell-autonomous, negative regulator of CM proliferation during zebrafish heart development. Our results highlight the role of SWI/SNF-mediated chromatin remodeling in developmental cardiac growth and suggest that Smarce1 may serve as an epigenetic modulator of cardiogenesis with relevance for future regenerative therapies.

## Introduction

The human heart is considered a terminally differentiated organ. In particular, adult human cardiomyocytes (CMs) are regarded as irreversibly post-mitotic, rendering them incapable of re-entering the cell cycle and proliferating to regenerate the myocardium, especially following myocardial injury. The molecular mechanisms driving the mitotic block in adult mammalian cardiomyocytes remain poorly understood, yet they hold significant therapeutic potential for enhancing and accelerating endogenous cardiac regenerative capacity following injury.

In contrast to humans, adult zebrafish possess a remarkable ability to fully regenerate their hearts, even after severe myocardial damage. This regenerative capacity is primarily driven by the high plasticity of zebrafish CMs, enabling their dedifferentiation, cell cycle re-entry, and proliferation to restore the injured myocardial tissue. Cardiac regeneration in zebrafish appears to largely depend on the reactivation of dormant developmental signaling pathways, including Jak/Stat signaling (Fang et al., 2013; Johnson et al., 2011), Gata (Kikuchi et al., 2010; Gupta et al., 2013; Jia et al., 2007; Singh et al., 2010), and T-box 20 signaling (Just et al., 2016a; Xiang et al., 2016). Notably, activation of the Tbx20 pathway in adult murine CMs after myocardial infarction enhances CM proliferation, leading to significantly improved functional cardiac repair (Xiang et al., 2016). Similarly, Chen et al. (2021) recently demonstrated that cardiac-specific overexpression of the developmental reprogramming gene cocktail OSKM (Oct4, Sox2, Klf4, and c-Myc) can overcome the mitotic block in adult murine CMs, thereby promoting endogenous cardiac regeneration

The cardiac transcription factors Tbx20, Gata4, and Nkx2–5 are known to interact with Brg1/Smarca4, an ATPase subunit of the SWI/SNF (SWI/sucrose non-fermentable) chromatin remodeling complex (Takeuchi et al., 2011). Myocardium-specific loss of Brg1 in mice results in early embryonic lethality due to myocardial growth defects, while Brg1-deficient zebrafish display severe cardiac hypoplasia (Bultman et al., 2000). In addition to Brg1, other members of the SWI/SNF complex, such as Baf60c (Lickert et al., 2004) or Baf180 (Wang et al., 2004), have also been identified as crucial regulators of cardiac development as their genetic ablation in mice leads to severe ventricular hypoplasia (Bevilacqua et al., 2014). These findings highlight the essential role of SWI/SNF chromatin remodeling components in heart growth and development.

In search of novel regulators of CM proliferation, we identified the zebrafish mutant *heart of stone* (*hos*), characterized by excessive cardiac growth due to the hyperproliferation of cardiomyocytes. We traced the loss-of-function mutation in *hos* to *smarce1*, a component of the SWI/SNF chromatin remodeling complex. Remarkably, myocardium-specific overexpression of *smarce1* suppressed proliferation in a cell-autonomous manner, identifying Smarce1 as a critical regulator of cardiomyocyte proliferation during heart development.

## Results

### Cardiac hyperplasia in zebrafish *heart of stone* mutant is due to increased proliferation rates

CM proliferation is fundamental for physiological heart growth during development and in the context of cardiac regeneration and repair (Foglia and Poss, 2016). In the past, the zebrafish model has successfully been used to identify novel components and signaling pathways that control heart growth during development and after injury (Takeuchi et al., 2011; Rottbauer et al., 2002; Buhler et al., 2021).

**FIGURE 1:**
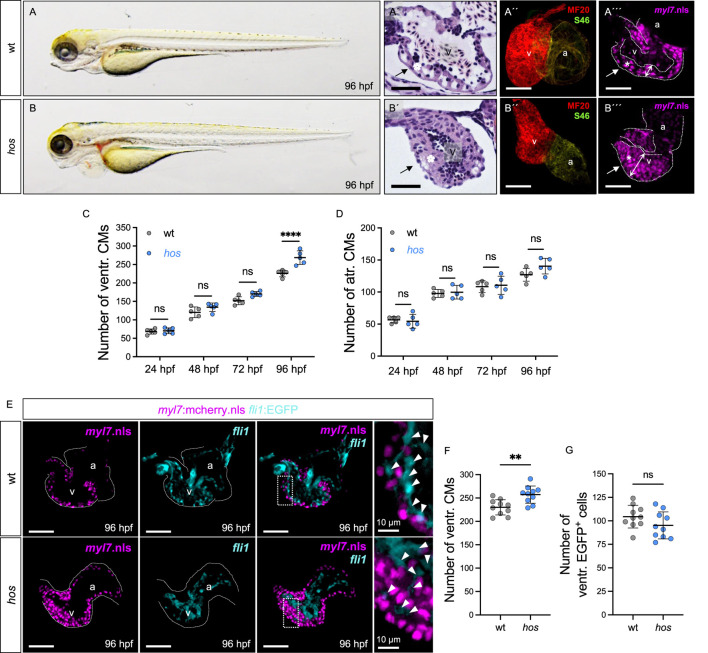
*Heart of stone* (*hos*) exhibits thickened myocardium accompanied with increased ventricular CMs. **(A,B)** Lateral view of wt clutch mates and *hos* mutant embryos at 96 hours post fertilization (hpf). **(A′,B′)** Hematoxylin/eosin staining of sagittal histological sections of wt and *hos* mutant ventricles at 96 hpf. In contrast to the wt ventricle, the *hos* mutant ventricular wall is thick and multilayered. **(A″,B″)** Confocal projections of wt and *hos* hearts dissected from Tg (*myl7*:mcherry.nls) and *hos* (*myl7*:mcherry.nls) at 96 hpf. **(A‴,B‴)** IF staining images of wt and *hos* hearts at 96 hpf. MF20 staining against meromyosin (red), which labels both cardiac chambers, the ventricle and atrium, and S46 staining against atrial-specific myosin (green) show normal cardiac chamber specification in both wt and *hos* embryos (scale bar: 50 µm). **(C,D)** Quantitative analyses of ventricular CMs at different developmental stages reveal significant increase in *hos* compared to that in wt at 96 hpf, while the *hos* mutation has no effect on the number of atrial CMs (n = 5). **(E)** Confocal images of dissected hearts from wt or *hos* crossed with Tg (*myl7*:cherry.nls;*fli1*:EGFP) at 96 hpf (scale bar: 50 µm). **(F,G)** Quantification of ventricular CMs and EGFP-positive (*fli1-*positive) cells in embryonic hearts of wt and *hos* at 96 hpf (n = 10). The number of endocardial cells in the ventricle is not altered in *hos*. v, ventricle; a, atrium; ventr., ventricular; atr., atrial.

In this study, we isolated and characterized the ENU-induced recessive embryonic-lethal zebrafish mutant *heart of stone* (*hos* ^*HJ163*^), which exhibits a massively thickened heart accompanied by ventricular chamber obliteration and reduced cardiac blood flow at 96 h post fertilization (hpf) (Figures 1A–B′), while cardiac defects were not observed at earlier developmental stages (Supplementary Figure S1). To determine whether the *hos* mutant underwent regular cardiac chamber specification and differentiation, we performed chamber-specific myosin heavy chain staining (MF20: ventricle and atrium, S46: atrium), demonstrating that the specification of chambers is normal in *hos* hearts (Figures 1A″–B″). To determine whether the thickening of the ventricular myocardium in *hos* mutants was due to increased CM numbers, which is termed cardiac hyperplasia, we isolated hearts from wild-type siblings (wt) and *hos* mutants crossed with Tg (*myl7*:mcherry.nls) transgenic fish showing nuclear mCherry fluorescence under the control of the CM-specific *myl7* promotor at various developmental stages and counted the mCherry-positive CMs of the ventricle (Figures 1A‴–B‴; Supplementary Figure S2). At 96 hpf, we found significantly increased numbers of CMs in *hos* ventricles compared to wt ventricles, whereas at earlier developmental stages, ventricular CM numbers and atrial CM numbers were not different between wt and *hos* mutants (Figures 1C, D). To assess whether the number of endocardial cells is also affected by *hos*, we crossed *hos* mutants with the transgenic myocardium (mCherry.nls) and endocardium (EGFP) reporter line Tg (*myl7*:mCherry.nls; *fli1*:EGFP) (Figure 1E) and specifically counted the endocardial cells in *hos* and wt controls. At 96 hpf, the number of EGFP^+^ endocardial cells was not altered (Figures 1F, G), indicating that increased cell proliferation is restricted to CMs in *hos* mutant zebrafish hearts.

**FIGURE 2:**
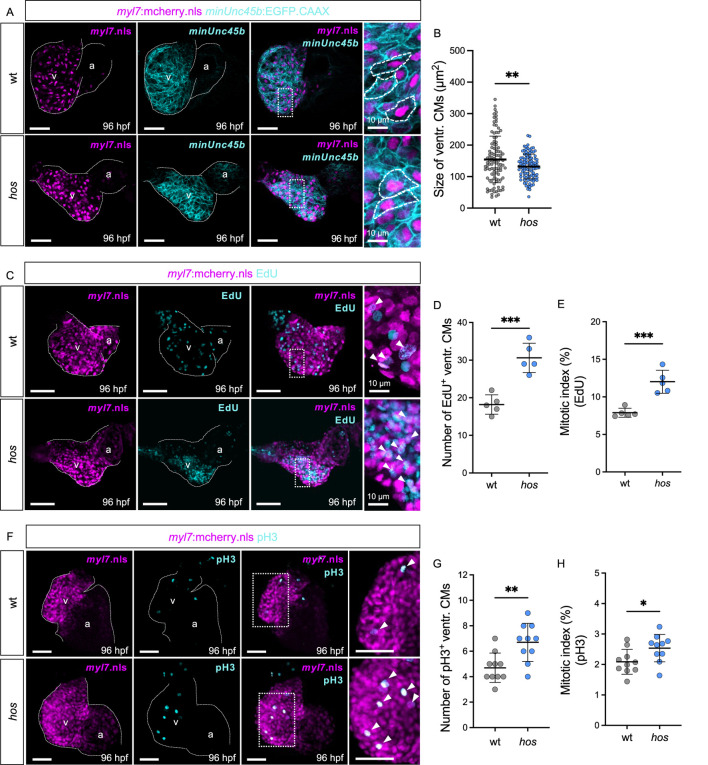
The *hos* mutation leads to not cardiac hypertrophy but hyperplasia due to increased proliferation of embryonic ventricular CMs. **(A)** Confocal projections of fluorescent CM nuclei (*myl7*.nls) and membrane (*minUnc45b*.CAAX) in embryonic hearts of wt and *hos* embryos at 96 hpf (scale bar: 50 µm). **(B)** Analyzed CM sizes of wt and *hos* ventricles (n = 102). **(C)** Dissected hearts of *hos* (*myl7*:mcherry.nls) incorporated with EdU visualizing DNA synthesis of cells at 96 hpf (scale bar: 50 µm). **(D,E)** Quantification of EdU-positive (EdU^+^) CMs indicating the S-phase of cell cycle **(D)** and the mitotic index of wt and *hos* **(E)** (n = 5). The *hos* mutation induces CM proliferative potential. **(F)** IF staining of pH3 visualizing the M-phase of cell proliferation in embryonic zebrafish hearts of wt and *hos* embryos at 96 hpf (scale bar: 50 µm). **(G,H)** Quantification of pH3-positive (pH3^+^) CMs and the mitotic index (pH3^+^ CMs/total ventr. CMs) in wt and *hos* ventricles at 96 hpf (n = 10). v, ventricle; a, atrium; ventr., ventricular.

To further investigate whether cardiac hypertrophy contributes to the thickening of the *hos* ventricle, we measured the CM size of wt and *hos* crossed with Tg (*myl7*:mCherry.nls; -*minUnc45b*:EGFP_CAAX) transgenic fish, in which the nuclei and cell membranes of the CM are labeled mCherry and EGFP, respectively (Figure 2A). The size of the ventricular CMs was not increased in *hos* but was even decreased compared to wt (Figure 2B), clearly demonstrating that the thickening of the ventricle in *hos* mutants is not due to their pathological hypertrophic enlargement but is exclusively caused by an increase in CM number. Next, to evaluate whether cardiac hyperplasia in *hos* is due to the increased proliferation rates of ventricular CMs, we conducted EdU incorporation experiments visualizing cells in the G1/S-phase of the cell cycle and phosphorylated histone 3 (pH3) immunostaining assays (M-phase marker) on dissected *hos* and wt control hearts at 96 hpf. As shown in Figure 2C, we found significantly increased numbers of EdU-positive (EdU^+^) ventricular CMs in *hos* mutants compared to age-matched wt controls, along with an increased mitotic index (EdU^+^ ventricular CMs/the total number of ventricular CMs) of CMs in *hos* ventricles (Figures 2D, E). Immunofluorescence (IF) staining of pH3 also confirmed an increased number of pH3-positive (pH3^+^) cells and a higher mitotic index of ventricular CMs in *hos* mutant hearts (Figures 2F–H). Additional pH3 analyses of embryonic stages at 24, 48, and 72 hpf showed no significant difference in proliferating CMs in the wt and *hos* ventricles (Supplementary Figure S3), implying a temporally restricted increase in CM proliferation in *hos* embryonic hearts.

In summary, our findings suggest that the pathological thickening of myocardial walls in *hos* mutant hearts is primarily driven by increased CM proliferation.

### *Heart of stone* encodes zebrafish SWI/SNF-related matrix-associated actin-dependent regulator of chromatin subfamily E member 1 (*smarce1*)

**FIGURE 3:**
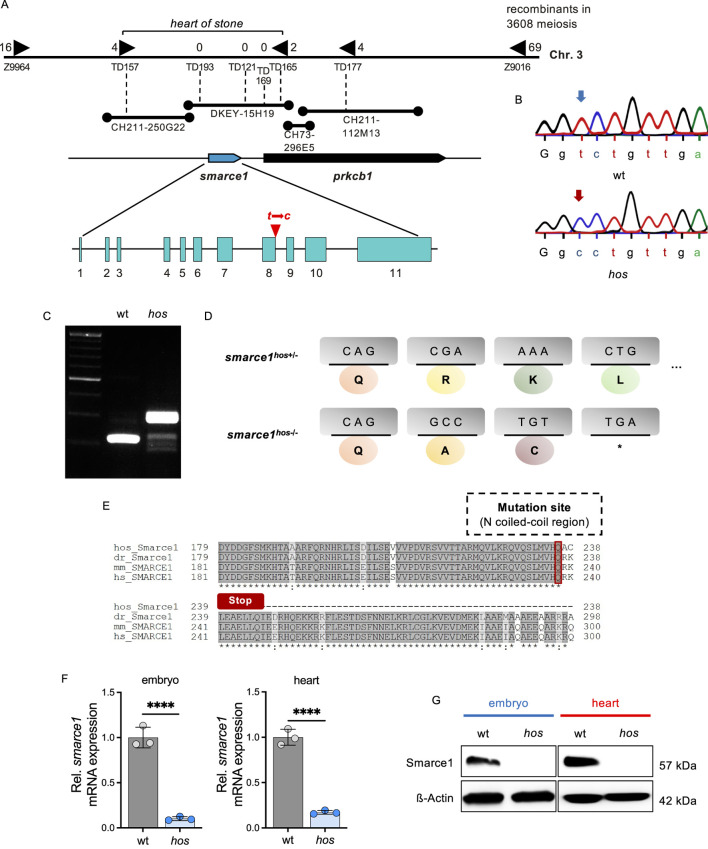
The *hos* encodes the SWI/SNF chromatin remodeling complex subfamily gene *smarce1*, resulting in a frame shift and premature stop of smarce1 translation. **(A)** Integrated genetic and physical map of the *hos* locus on zebrafish chromosome 3. The *hos* mutation interval is flanked by the microsatellite markers Z9964 and Z9016 and was further defined using custom-made microsatellite markers (TD). The final *hos* interval encodes one full open reading frame, zebrafish *smarce1*, and an additional partial open reading frame, zebrafish prkcb1. Sequencing of both genes reveals a point mutation of thymine to cytosine at the splice donor site of intron 8 of the *smarce1* gene. **(B)** Sequencing results showing a point mutation of thymine to cytosine at a splice donor site (intron 8) of *smarce1* in *hos*. **(C)** Amplified *smarce1* targeting intron 8 by RT-PCR, indicating an intron inclusion resulting from the point mutation. **(D)** Schematic description of translational premature stop codon (*) by the *hos* point mutation. **(E)** Smarce1 protein sequence alignment of homozygous *hos* mutant, wild-type zebrafish, mouse, and human nearby mutation site (dr: *Danio rerio*; mm: *Mus musculus*; hs: *Homo sapiens*). **(F)** Relative *smarce1* mRNA expression in *hos* embryo and heart compared to that in wt at 96 hpf (n = 3). **(G)** Smarce1 protein levels are not detectable in *hos* embryo and heart compared to that in wt controls at 96 hpf.

In search of the ENU-induced genetic mutation causing accelerated embryonic CM proliferation in *hos*, a genome-wide study of microsatellite marker segregation was performed, and *hos* was linked to zebrafish chromosome 3. Recombination analysis of 1,804 *hos* mutant embryos and genetic fine-mapping restricted *hos* to a genomic interval including the zebrafish SWI/SNF-related matrix-associated actin-dependent regulator of chromatin subfamily E member 1 (*smarce1/Baf57*) and the protein kinase C beta 1 (*prkcb1*) genes (Figure 3A). We sequenced the entire coding sequences of *smarce1* and *prkcb1*, along with all the exon–intron/intron–exon boundaries of zebrafish *smarce1* from wild-type and *hos* mutant genomic DNA. Thus, we identified the *hos* mutation to be a point mutation substituting thymine for cytosine in the splice donor site of intron 8 of *smarce1*, which is predicted to cause defective splicing and the integration of intron 8 into the *smarce1* mRNA (Figures 3B,C).

Intron 8 integration into *smarce1* mRNA is predicted to either mitigate nonsense-mediated RNA decay or disrupt the regular reading frame with the generation of a premature stop codon and, subsequently, the premature termination of protein translation (Figures 3D, E). Hence, to evaluate the effect of the *hos* mutation on *smarce1* RNA stability, we performed *smarce1*-specific quantitative real-time PCR (qRT-PCR) analyses on mRNAs extracted from whole embryos and the dissected embryonic hearts of wt and *hos* mutants at 96 hpf and found that *smarce1* mRNA levels were significantly reduced in *hos* mutant samples compared to samples derived from age-matched wild-type siblings (Figure 3F), suggesting that the *hos* mutation induces nonsense-mediated *hos* mutant *smarce1* mRNA decay (NMD). Accordingly, Western blot analyses using whole-embryo or heart-specific protein lysates and a Smarce1-specific antibody directed against the N-terminus of the Smarce1 protein severely reduced Smarce1 protein levels in *hos* mutant samples compared to control protein lysates (Figure 3G), whereas a truncated form of Smarce1 could not be detected in *hos* samples. These findings suggest that the identified splice donor site mutation in *hos* results in the degradation of *hos* mutant mRNA and, finally, in the loss of Smarce1 protein expression.

### Targeted knockdown of Smarce1 in wild-type zebrafish increases embryonic cardiomyocyte proliferation

**FIGURE 4:**
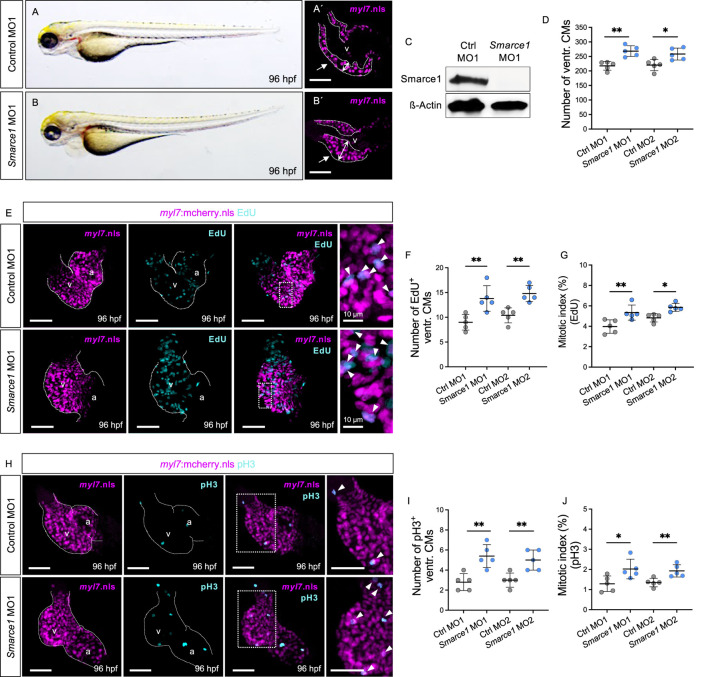
Knockdown of zebrafish smarce1 phenocopies *hos* cardiac hyperplasia. **(A,B)** Lateral views of control morpholino (MO; ctrl MO1) or *smarce1* start MO (*smarce1* MO1)-injected embryo at 96 hpf. Knockdown of smarce1 phenocopies the *hos* mutant phenotype, whereas the injection of specific control MO does not. **(A′,B′)** Dissected hearts from Tg (*myl7*:mcherry.nls) embryos after control or *smarce1* MO1 injection (scale bar: 50 µm). Smarce1 knockdown results in a thickened ventricular wall of developing zebrafish heart at 96 hpf. **(C)** Smarce1 protein is absent in *smarce1* MO1-injected embryos at 96 hpf. **(D)** Quantitative analyses of ventricular CM numbers show a significant increase in the hearts of *smarce1* MO1- or MO2-injected embryos at 96 hpf (n = 5). **(E)** Confocal images of dissected hearts from control- or *smarce1* MO1-injected embryos with EdU incorporation displaying CM nucleus (mCherry) and proliferating CMs (cyan) at 96 hpf (scale bar: 50 µm). **(F,G)** Numbers of EdU^+^ CMs and the mitotic index are significantly enhanced in *smarce1* MO-injected embryonic ventricles compared to control (ctrl) MO-injected hearts at 96 hpf (n = 5). **(H)** IF images of *smarce1* morphant (MO1) heart with pH3 staining displaying CM nucleus (mCherry) and proliferating CMs (cyan) at 96 hpf (scale bar: 50 µm). **(I,J)** Statistical assessment of counting pH3^+^ ventricular CMs and the mitotic index in wt and *hos* at 96 hpf after the injection of specific control MO1/2 or *smarce1* MO1/2 reveals increased proliferation in zebrafish embryonic ventricles by the knockdown of smarce1(n = 5). v, ventricle; a, atrium; ventr., ventricular.

Next, to substantiate that ventricular hyperplasia in *hos* mutant zebrafish is due to the loss of zebrafish Smarce1 function, we knocked down Smarce1 by injecting morpholino-modified antisense oligonucleotides (MOs) directed either against the translational start site (*smarce1* MO1) or the splice donor site of exon 8/intron 8 (*smarce1* MO2) into wild-type zebrafish embryos at the one-cell stage (Figures 4A, B). By Western blot analyses, we confirmed the high knockdown efficacy of the used *smarce1* morpholinos (Figure 4C; Supplementary Figure S4A). Smarce1 morphants displayed severely thickened ventricular myocardium, leading to an obliterated ventricular lumen (Figures 4A′–B′). Similar to the situation in *hos* mutant hearts, at 96 hpf, we also found significantly higher amounts of CMs in Smarce1 morphant ventricles than in control-injected embryos (Figure 4D). In addition, EdU incorporation assays (Figure 4E; Supplementary Figure S4B) revealed significantly increased numbers and proportions of EdU^+^ ventricular CMs (Figures 4F, G) in Smarce1 morphants. Similarly, pH3 staining (Figure 4H; Supplementary Figure S4C) demonstrated a marked increase in the number and proportion of pH3^+^ CMs in Smarce1 morphant hearts compared to hearts injected with control MOs (Figures 4I, J). These findings confirm that the loss of Smarce1 function induces cardiac hyperplasia in the zebrafish heart.

### Restoration of *smarce1* mRNA rescues increased cardiomyocyte proliferation in *hos* embryos

**FIGURE 5:**
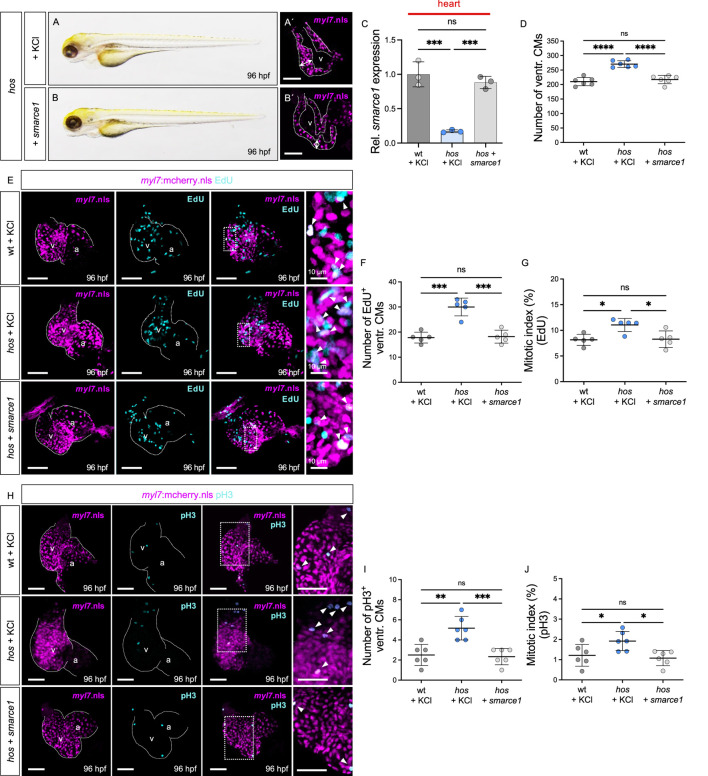
Restoration of *smarce1* mRNA suppresses excessive CM proliferation. **(A,B)** Lateral view of *hos* mutant embryos injected with KCl (control) or wt zebrafish *smarce1* mRNA at 96 hpf. **(A′,B′)** Confocal images of *hos* (*myl7*:mcherry.nls) with KCl or *smarce1* mRNA injection at 96 hpf (scale bar: 50 µm). **(C)** Relative *smarce1* mRNA expression in the hearts of wt or *hos* embryos with KCl or *smarce1* mRNA injection at 96 hpf (n = 3). **(D)** Number of ventricular CMs in wt or *hos* mutants with injection of KCl or *smarce1* mRNA at 96 hpf. **(E)** Confocal images of EdU-incorporated hearts dissected from Tg (*myl7*:mCherry.nls) or *hos* (*myl7*:mCherry.nls) with KCl or *smarce1* mRNA injection (scale bar: 50 µm). **(F,G)** Statistical analyses of the EdU assay (EdU^+^ ventricular CMs and the mitotic index) in KCl- or *smarce1* mRNA-injected wt and *hos* (n = 5). Injection of *smarce1* mRNA rescues cardiac hyperplasia of *hos*. **(H)** Confocal images of dissected hearts from wt or *hos* crossing with Tg (*myl7*:mcherry.nls) after KCl or *smarce1* mRNA injection (scale bar: 50 µm). IF staining of pH3 visualizes proliferating cells in the hearts of MO-injected embryos at 96 hpf. **(I,J)** Quantification of pH3^+^ CMs and the mitotic index showing attenuated ventricular CM proliferation in *hos* by *smarce1* mRNA injection at 96 hpf (n = 5). v, ventricle; a, atrium; ventr., ventricular.

Next, to evaluate whether the reconstitution of *smarce1* expression can preserve physiological heart growth by normalizing CM proliferation in *hos* mutants, we overexpressed wild-type *smarce1* mRNA in homozygous mutant *hos* embryos (Figures 5A–B′). Injection of *smarce1* mRNA normalized the reduced *smarce1* mRNA expression in *hos* hearts to levels comparable to those in wt hearts. (Figure 5C). The total ventricular CM numbers, EdU- and pH3-positive CM numbers, and the mitotic index of *smarce1* mRNA-injected *hos* mutants were comparable to those of KCl-injected wt and significantly decreased compared to KCl-injected *hos* mutants at 96 hpf (Figures 5D–J). These findings demonstrate that the overexpression of wild-type *smarce1* mRNA in *hos* mutant embryos can control and reduce CM proliferation rates to normal levels.

### Myocardial-specific overexpression of *smarce1* impairs embryonic cardiomyocyte proliferation

**FIGURE 6:**
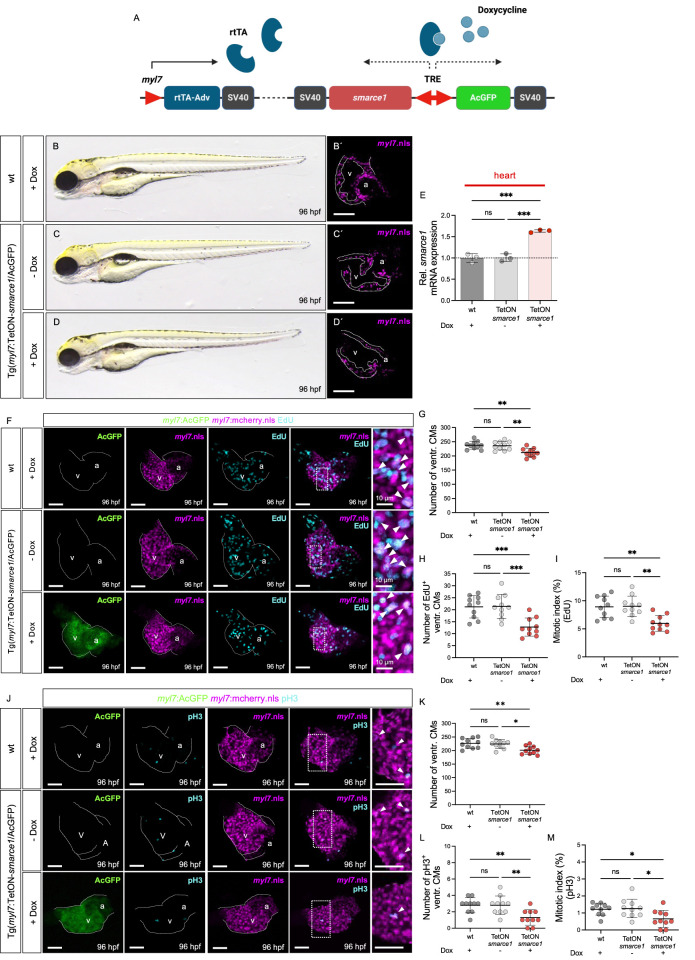
Myocardium-specific *smarce1* overexpression reduces ventricular CM proliferation in the embryonic heart of Tg (*myl7*:Tet-On-s*marce1*/AcGFP). **(A)** Illustration of Tet-On system structure. The protein of rtTA-Adv is specifically expressed in the myocardium by the *myl7* promotor. Under doxycycline treatment, *smarce1* and AcGFP are bi-directionally induced in the myocardium. **(B–D)** Lateral view of wt and Tg(*myl7*:Tet-On-*smarce1*/AcGFP) embryos with or without doxycycline (dox) treatment at 96 hpf. **(B′–D′)** Confocal images of dissected hearts from Tg(*myl7*:mCherry.nls) and Tg(*myl7*:Tet-On-*smarce1*/AcGFP) × Tg(*myl7*:mCherry.nls) with or without dox treatment at 96 hpf. **(E)** Transcriptional level of *smarce1* in the hearts of wt or Tg (*myl7*:Tet-On-*smarce1*/AcGFP) embryos showing dox-induced *smarce1* overexpression at 96 hpf. **(F)** Confocal IF images of wt and Tg (*myl7*:Tet-On-*smarce1*/AcGFP) embryonic hearts with CM nuclei (*myl7*:mCherry.nls) and EdU incorporation at 96 hpf (scale bar: 50 µm). **(G–I)** Quantitative analyses of ventricular CM numbers, EdU^+^ ventricular CMs, and the mitotic index in wt or *smarce1*-overexpressed developing hearts at 96 hpf (n = 10). **(J)** IF images of the hearts from Tg (*myl7*:mCherry.nls) and Tg (*myl7*:Tet-ON-*smarce1*/AcGFP) × Tg(*myl7*:mCherry.nls) embryos stained with pH3 (scale bar: 50 µm). **(K–M)** Quantitative analyses of ventricular CM numbers, pH3^+^ ventricular CMs, and the mitotic index at 96 hpf (n = 10). v, ventricle; a, atrium; ventr., ventricular.

To further delineate the role of Smarce1 in regulating CM proliferation, we generated a transgenic Tet-On system enabling myocardium-specific and temporally controlled *smarce1* expression in zebrafish (Figure 6A). Embryos were exposed to doxycycline from 0 to 96 hpf to induce *smarce1* overexpression in the CMs. Heart-specific qRT-PCR confirmed the robust induction of *smarce1* expression in Tg (*myl7*:Tet-On-*smarce1*/AcGFP) embryos following doxycycline treatment (Figure 6E). Importantly, myocardial *smarce1* overexpression did not elicit gross morphological cardiac defects compared to doxycycline-treated wild-type siblings or uninduced transgenic controls (Figures 6A–D′).

**FIGURE 7:**
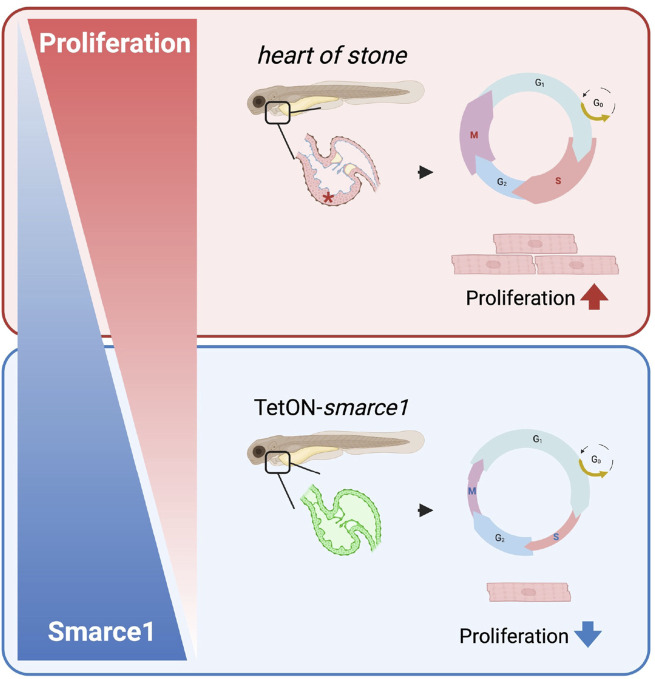
Schematic illustration showing the regulatory role of smarce1 in CM proliferation of embryonic zebrafish heart. Smarce1 negatively regulates CM proliferation in the embryonic zebrafish heart as its loss (*hos*) leads to cardiac hyperplasia by increasing CM proliferation, while its overexpression (Tet-On-*smarce1*) suppresses CM proliferation.

To assess the functional consequences of *smarce1* overexpression on CM proliferation, we conducted EdU incorporation assays and pH3 staining at 96 hpf (Figures 6F, J). Quantitative analysis of ventricular CM numbers, EdU- and pH3-positive nuclei, and the mitotic index revealed a significant reduction in CM proliferation in *smarce1*-overexpressing embryos relative to wild-type controls (Figures 6G–I, K–M). These results establish Smarce1 as a cell-autonomous, negative regulator of cardiomyocyte proliferation during zebrafish heart development (Figure 7).

## Discussion

In search of novel molecular programs that guide CM proliferation, in this study, we characterized the zebrafish cardiac hyperplasia mutant *hos* and found that the SWI/SNF chromatin remodeling complex subunit Smarce1 is a key regulator of CM proliferation in embryonic heart development.

Smarce1 is a component of the SWI/SNF chromatin remodeling complex, which is known to play an important role in cell differentiation, cell maturation, and transcriptional regulation (Centore et al., 2020; Ho and Crabtree, 2010). The mammalian SWI/SNF chromatin remodeling complex, also known as the Brg1/Brm-associated factor (Baf) complex, contains numerous subunits encoded by at least 28 genes, including one of the two catalytic subunits with ATPase activity, Brg1 or BRM. The Smarce1/Baf57 subunit is present in all higher eukaryotic SWI/SNF chromatin remodeling complexes but is absent in yeast (Lomeli and Castillo-Robles, 2016), implying that Smarce1 might contribute to specialized SWI/SNF chromatin remodeling complex functions in higher animals. Tight regulation and sensing of Smarce1 levels appear to be crucial for the overall stoichiometry of the SWI/SNF chromatin remodeling complex and, thus, for its function (Chen and Archer, 2005).

The SWI/SNF complex is a known master regulator of cell growth and proliferation, acting either as an activator (Hah et al., 2010) or as a suppressor (Xia et al., 2008), depending on context-specific situations or modulation by specific SWI/SNF complex subunits (Lomeli and Castillo-Robles, 2016). The loss of certain subunits of the SWI/SNF chromatin remodeling complex in zebrafish, such as Brg1 or Baf45c, resulted in severe cardiac abnormalities, including thin and hypoplastic myocardial cell layers (Lange et al., 2008; Takeuchi et al., 2011). Brg1 was also found to be a positive regulator of cardiac regeneration by ensuring CM proliferation in adult zebrafish hearts after injury through the control of cell cycle-dependent kinase inhibitors such as *cdkn1a* and *cdkn1c* (Xiao et al., 2016). In mice, CM-specific deletion of Brg1 also resulted in a thin, compact myocardium due to impaired CM proliferation (Hang et al., 2010). In contrast to the situation in Brg1-deficient zebrafish embryos, we found that deficiency of Smarce1 leads to enhanced CM proliferation without affecting hypertrophic growth, whereas overexpression of Smarce1 impairs CM proliferation in zebrafish, indicating that Smarce1, unlike Brg1, negatively regulates SWI/SNF chromatin remodeling complex function and CM proliferation (Takeuchi et al., 2011) and that fine-tuned smarce1 levels are essential for the regular and physiological proliferation of CMs. Interestingly, several other SWI/SNF components were recently found to be involved in the control of CM proliferation. For example, Baf60c induces CM proliferation in newborn mice (Lickert et al., 2004; Sun et al., 2018), while the component Baf250a controls CM proliferation and differentiation by regulating the recruitment of Oct4 and β-catenin to the Ccnd2 and Ccnd3 promoters, two key genes that are fundamental for S-phase entry during cell cycle progression in human embryonic stem cells (Lei et al., 2019).

We have shown in this study that the loss of Smarce1 in the zebrafish mutant *hos* leads to increased CM proliferation during heart development, resulting in severe hyperplasia of the heart. Interestingly, no increased CM proliferation has been reported in zebrafish with targeted CRISPR/Cas9-mediated silencing of Smarce1, although extreme ventricular compaction has been described (Castillo-Robles et al., 2018). The phenotypic divergence between the ENU-derived *hos* allele and the CRISPR/Cas9 *smarce1* knockout likely reflects more than the simple loss of Smarce1 expression. Although both models abolish *smarce1* mRNA and protein, the intron-8 point mutation in *hos* versus the small exon-4 deletion introduced by CRISPR may engage distinct post-transcriptional and compensatory mechanisms. In particular, intronic lesions can alter splicing patterns or produce truncated peptides with dominant-negative activity, whereas exon frameshifts more reliably provoke nonsense-mediated mRNA decay (NMD) and, as a result, transcriptional adaptation via the upregulation of homologous loci (Junker, 2019; Anderson et al., 2017), as observed in several other CRISPR/Cas9-mediated mutant zebrafish lines (Diofano et al., 2020; El-Brolosy et al., 2019; El-Brolosy and Stainier, 2017). Moreover, subtle differences in genetic background—including strain-specific modifier alleles—and the variable persistence of maternally deposited *smarce1* transcripts or protein could buffer one mutant line more effectively than the other. Finally, potential CRISPR off-target events or mosaicism might partially rescue Smarce1 function in a subset of cells (Zhang et al., 2015). Disentangling these possibilities will require direct, side-by-side analyses of transcript splicing, NMD efficiency, compensatory gene expression, and precise protein-truncation patterns in both mutant lines. Meanwhile, Castillo-Robles et al. (2018) found that Smarce1 binds to the cis-regulatory regions of the *Gata5* transcription factor gene, and its CRISPR/Cas9-mediated loss leads to a significant upregulation of *Gata5* transcription 4 days after fertilization. Interestingly, Gata5 itself is known to promote CM proliferation during zebrafish embryogenesis, particularly due to its regulatory interplay with the Notch signaling target *hey2*/*gridlock* (Jia et al., 2007). In addition, Gata5 also regulates CM proliferation in mammals and during heart regeneration in zebrafish (Kikuchi et al., 2011; Singh et al., 2010).

In HeLa cells, SMARCE1 also appears to be involved in cell cycle regulation, as ablation of SMARCE1 function resulted in decreased cell proliferation due to transcriptional downregulation of key cell cycle regulators (Hah et al., 2010). Furthermore, SMARCE1 has been implicated in the induction of cell cycle arrest in the breast cancer cell line BT549, which expresses truncated smarce1 proteins (Wang et al., 2005). In this study, the rescued protein content of wild-type SMARCE1 resulted in cell cycle arrest at the G2-M phase caused by the decrease in cyclin E1 and Cdc25A. Although, as described above, other SWI/SNF components such as Brg1 and Baf60c (Lickert et al., 2004; Takeuchi et al., 2011) have already been shown to play an essential role in the CM cell cycle, it will be interesting to investigate the *in vivo* role of SMARCE1 in the developing and adult mammalian heart in future studies. In this context, SMARCE1 is far more than a static ‘building block’—it dynamically directs the SWI/SNF complex to specific genomic loci, thereby fine-tuning gene expression programs. Based on our data, we propose that SMARCE1 exerts a repressive influence on the activity of the SWI/SNF complex. Our findings show that the loss of SMARCE1 function leads to markedly increased cardiac growth, driven by significantly enhanced cardiomyocyte proliferation. This suggests that SMARCE1 acts as a negative regulator within the SWI/SNF complex, at least in cardiomyocytes, which is a novel and unexpected role. Further studies will be needed to elucidate the precise molecular mechanisms underlying this SMARCE1-dependent control of cardiomyocyte proliferation. Results that support this hypothesis have already been shown in other cell populations but not in CMs. For instance, SMARCE1 stabilizes the canonical Baf (SWI/SNF) chromatin-remodeling complex. Its loss disrupts chromatin accessibility and unleashes pro-proliferative gene programs, promoting aggressive tumor growth, such as clear-cell meningioma (St Pierre et al., 2022). Furthermore, SMARCE1 is essential for organized mitosis in embryonic stem cells. The loss of smarce1 disrupts both epigenetic and genetic regulatory mechanisms, which, in certain settings, can facilitate unchecked cell division (Zhu et al., 2023).

Recent findings in animal models that exhibit a high regenerative capacity of the heart, such as zebrafish or neonatal mice, suggest that signaling pathways and mechanisms critical for the control of developmental CM proliferation may be promising targets for the activation of CM proliferation and regeneration in the adult mammalian heart. In this context, epigenetic regulators and/or proteins that control chromatin remodeling have recently been discovered as fundamental regulators of CM proliferation. For example, a deficiency of histone deacetylase 1 (Hdac1), which is known to be significantly involved in the epigenetic control of gene transcription and chromatin remodeling, was found to lead to impaired CM proliferation in the embryonic and also in the adult zebrafish heart after cryoinjury (Buhler et al., 2021). Similarly, Reptin and Pontin, two components of repressive polycomb complex 1 (PRC1) and known chromatin remodelers, have been associated with significantly increased embryonic CM proliferation in zebrafish (Rottbauer et al., 2002; Sharpe et al., 2022). Interestingly, Reptin interacts directly with Hdac1 (Kim et al., 2005; Mikesch et al., 2018), suggesting that CM proliferation, which controls chromatin remodeling, is fine-tuned and coordinated by different but cooperating chromatin remodeling complexes such as SWI/SNF, polycomb, and Hdac.

In summary, our study demonstrates that fine-tuned regulation of the SWI/SNF chromatin remodeling subunit Smarce1 orchestrates cardiomyocyte proliferation in the embryonic zebrafish heart. Further investigations employing epigenetic and transcriptomic profiling are required to elucidate the precise mechanisms underlying Smarce1 function. Additionally, exploring whether SWI/SNF-mediated signaling can overcome the mitotic block in adult cardiomyocytes may provide novel insights into its potential role in cardiac regeneration in zebrafish and mammals.

## Materials and methods

### Animals

All procedures and experiments in this study were carried out after obtaining appropriate institutional approvals (Tierforschungszentrum (TFZ) Ulm University; No. 0183, 24.03.2011; Regierungspräsidium Tübingen; No. 1415) and according to the national (Germany) ethical and animal welfare regulation (Tierschutzgesetz § 11). All experimental procedures in this study were performed according to the guidelines from the EU Directive 2010/63/EU on the protection of animals used for scientific purposes. The care and breeding of zebrafish (*Danio rerio*) were carried out as previously described (Just et al., 2016b). For injection experiments, the TüAB wild-type strain was used. Adult *hos* mutants were kept as heterozygous fish, and breeding resulted in 25% homozygous *hos* mutant offspring. Pictures or movies were recorded at 24, 48, 72, and 96 h post fertilization (hpf). For documentation, zebrafish embryos were treated with 0.003% 1-phenyl-2-thiourea to inhibit pigmentation. For immunofluorescence or heart-specific analyses, the Tg (*myl7*:mCherry.nls), Tg (*myl7*:GFP), Tg (*myl7*:mCherry.nls;*fli1*:EGFP), Tg (*minUnc45b*:EGFP.CAAX), or Tg (*myl7*:mCherry-CAAX) lines were used, marking cardiomyocytes with mCherry or GFP expression.

### Positional cloning

DNA from 48 *hos* mutants and 48 wild-type siblings was pooled, and bulked segregation analysis was performed as described before (Buhler et al., 2021). *Hos* was located on chromosome 3, and the critical genomic interval for *hos* was defined by genotyping 1,804 mutant embryos for polymorphic markers in the area. The *hos* locus was restricted to two overlapping bacterial artificial chromosomes (BACs), zCH211-250G22 and zDKEY-15H19. Further recombination analyses using single-nucleotide polymorphisms (SNPs) and simple sequence-length polymorphisms (SSLPs) derived from the sequence of overlapping BAC clones allowed the *hos* mutation interval to be restricted to a 139.7 kb region on BAC zDKEY-15H19; this region contains two open reading frames encoding the proteins SWI/SNF-related, matrix-associated, actin-dependent regulator of chromatin, subfamily e, member 1 (*smarce1*; NP_958455.2) and protein kinase C, beta b (*prkcb1*; NP_957272.1).

### Sequencing the genomic region of mutation

Genomic DNA (gDNA) from wild-type embryos and *hos* mutants was extracted by incubation with DNA lysis buffer (50 mM KCl, 0.3% Tween 20, 0.3% NP40, and 10 mM Tris/HCl; pH 8.3) including proteinase K (620 µg/mL) overnight at 50 °C. The gDNA was amplified, inserted into the TOPO cloning vector, sequenced, and analyzed to compare the readout between wild-type and mutant alleles.

### Microinjection

Microinjections were performed at the one-cell stage into fertilized zebrafish oocytes using pulled glass capillaries and a microinjector. Embryos were then allowed to develop at 28.5 °C until the indicated stages. To inhibit pigmentation, 0.003% 1-phenyl-2-thiourea was added to the regular embryo medium E3 (5 mM NaCl, 0.17 mM KCl, 0.33 mM CaCl_2_, and 0.33 mM MgSO_4_ dissolved in water). Morpholino-modified antisense oligonucleotides (MOs; Gene Tools, LLC) were injected into one-cell stage zebrafish embryos. To knockdown zebrafish Smarce1, MO targeting the translational start site (*smarce1*-MO1: 5′-GCCGCTTTGACATCTTGATTGTAGG-3′) or the splice donor site of zebrafish *smarce1* (*smarce1*-MO2: 5′-TTGATATGCTCAACAGACCTGGTGC) or the 5 base pair missense MOs as the control (control-MO1: 5′-GtCGCTTcGACgTCTTGATTaTAcG-3′, control-MO2: 5′-TcGATATGCTCtACAGAtCTcGTGg-3′) were injected into fertilized oocytes at the one-cell stage. MOs were injected with 2.5 ng in 0.2 M potassium chloride. For rescue experiments, zebrafish *smarce1* cDNA was amplified using Q5® High-Fidelity DNA Polymerase and cloned into the donor plasmid (pDONR221) and destination vector (pDestCS2+) using the Gateway Cloning System. Capped RNA of zebrafish *smarce1* was synthesized from *smarce1* cDNA-inserted pCS2+ vector using the mMESSAGE mMACHINE System, and 0.25 ng of mRNA in 0.2 M potassium chloride was injected.

### RNA extraction and quantitative real-time PCR

Per biological replicate, a pool of 25 embryos and 100 embryonic hearts was collected at 96 hpf. RNA extraction was carried out using an RNeasy Mini Kit, according to the manufacturer’s instructions. Total RNA (200 ng) was reverse-transcribed to produce cDNA using Superscript III Reverse Transcriptase. Quantitative real-time PCR was carried out according to the standard protocols using SYBR Green on a LightCycler 480 II. Two housekeeping genes, *β-actin* and *18S ribosomal RNA*, were used as reference genes for normalization of gene expression (list of primer sequences; Supplementary Table S2).

### Protein lysate extraction and Western blot analysis

For each independent Western blot experiment, 50 embryos at 96 hpf or 100 embryonic hearts expressing GFP exclusively in cardiomyocytes (Tg(*myl7*:GFP)) dissected at 96 hpf from wild-type siblings and *hos* mutant zebrafish were used, respectively. Whole embryos were dechorionated manually, and the yolk was removed using the deyolking buffer (55 mM NaCl, 1.8 mM KCl, and 1.25 mM NaHCO_3_). Deyolked embryos were washed with washing buffer (110 mM NaCl, 3.5 mM KCl, 2.7 mM CaCl2, and 10 mM Tris/HCl at pH 8.5) 3–4 times. Embryos were resuspended in the RIPA buffer (10 mM Tris, 150 mM NaCl, 0.5 mM EDTA, 0.1% SDS, 1% Triton X-100, and 1% sodium deoxycholate) containing protease inhibitor (cOmplete™) and phosphatase inhibitor (PhosSTOP™) and frozen in liquid nitrogen for manual homogenization with pestles. Homogenized embryos were incubated on ice for 30 min and centrifuged for 15 min at 18,000 *g* and 4 °C. The supernatant was collected and measured using the Bradford assay. For Western blot analysis, protein lysates were boiled in 5× (protein lysate of whole mount embryos or mice hearts) or 2× (embryonic heart tissues) Laemmli buffer and loaded on a precast 8%–16% SDS gel. Proteins were separated by SDS-PAGE and transferred to a polyvinylidene fluoride (PVDF) membrane. After blocking in 5% skim milk powder in TBST (TBS with 0.1% Triton) for 2 h at room temperature (RT), the membrane was incubated with the primary antibodies overnight at 4 °C. The primary antibodies used are listed in Supplementary Table S1. The corresponding anti-rabbit or -mouse IgG HRP-linked secondary antibodies were incubated for 2 h at RT after washing with TBST. Signals were detected by chemiluminescence using a luminescent image analyzer (ImageQuant LAS 4000 mini). Western blots were quantified using ImageQuant LAS 4000 software and normalized to β-actin of each protein target and wt sibling.

### EdU incorporation and immunofluorescence staining

For EdU incorporation assays in zebrafish embryo hearts, the Click-iT™ EdU Alexa Fluor™ 488 or 647 Imaging Kit was used. Embryos at 96 hpf were pulsed for 2 h with EdU on ice and fixed with 4% paraformaldehyde (PFA) in PBS overnight at 4 °C. After fixation, hearts were dissected using a 30 G syringe needle and permeabilized in 1% Triton X-100 PBS before staining with EdU reaction cocktail. To perform pH3 staining, embryos were prepared at the corresponding time point and fixed in 4% PFA overnight at 4 °C. Afterward, samples were permeabilized in 2.5 mg/mL trypsin in 0.1% Triton in PBS (PBT) for 5 min on ice and blocked in 5% normal goat serum (NGS) in PBT for 1 h at RT. The primary antibody, anti-phospho-histone H3 (Ser10) (Millipore, Cat #06-570; 1:100), was incubated overnight at 4 °C, followed by the corresponding Alexa Flour 488- or -647-conjugated secondary antibody. All samples were mounted using VECTASHIELD® HardSet™ (with DAPI) for imaging.

### Histology

For histology, embryos were fixed in 4% PFA (in PBS), embedded in JB-4, cut into 5 μm sections using a Leica RM2255 microtome, dried, and stained with hematoxylin and eosin. The sections were visualized using Axioskop 2 plus.

### Imaging and counting cardiomyocytes

Whole-mount zebrafish embryo images were taken using the Olympus SZX 16 microscope after orientation in 2.5% methyl cellulose. Fluorescent pictures of the fluorescent cardiomyocytes were taken using the Leica DMi8 confocal microscope with a ×40 objective (with oil). The total number of cardiomyocytes in the entire ventricle or atrium was quantified. Hearts were dissected, and high-resolution confocal z-stacks were acquired at 0.44 µm intervals; every optical stack was then used for cell counting. For the confocal projections shown in the figures, the full z-stack was merged. This ensures that rare pH3- or EdU-positive cardiomyocytes, which are often missed in individual stacks, are accurately visualized and represented. CM numbers were counted using the ImageJ cell counter plugin (particle analysis; point picker) and recounted for verification using Imaris (Oxford Instruments). For statistics, the data were analyzed and visualized using GraphPad Prism 9.

### Generation of *smarce1*-inducible zebrafish models

To establish Tg (*myl7*:Tet-ON-*smarce1*/AcGFP), the vectors including zebrafish *smarce1*, *Aequorea coerulescens*-derived green fluorescence protein (AcGFP), the trans-activator protein, and a modified tetracycline response element (TRE) cDNA were integrated into a destination vector for the zebrafish (pDestCS2+) through the process of gateway cloning. The final plasmid was injected into zebrafish embryos at the one-cell stage, and the resulting fish were maintained to establish a homozygous line.

### Statistical analysis

All graphs and statistical analyses are expressed as the means ± standard deviation (SD), and analyses were performed using a one-way ANOVA or two-way ANOVA. When necessary, data were expressed as the means ± SD of at least three independent experiments, and statistical analysis for single comparison was performed using the Student’s t-test (Mann–Whitney test). Statistical significance was determined using the Holm–Sidak method, and a value of *p* < 0.05 was accepted as statistically significant.

## Data Availability

The original contributions presented in the study are included in the article/Supplementary Material, further inquiries can be directed to the corresponding author.

## Supplementary material

SUPPLEMENTARY FIGURES1 Heart of stone (hos) mutation induces no phenotypic defects at early developmental stages. **(A)** Lateral views of wild type (wt) and heart of stone (hos) mutant embryos at 24, 48, 72 hours post fertilization (hpf). The embryonic phenotype of hos mutant is comparable to that of wt embryos up to 72 hpf.

SUPPLEMENTARY FIGURES2 The hos mutation causes abnormal embryonic heart growth at late developmental time. **(A)** Dissected hearts of wt and hos crossing with Tg(myl7.mcehrry.nls) at different developmental stages (scale bar: 50 ?m).

SUPPLEMENTARY FIGURES3 Proliferation of ventricular CMs is not affected at early embryonic stages in hos. **(A)** IF staining against pH3 (cyan) in dissected hearts of wt or hos crossing with Tg(myl7:mcherry.nls) at 24, 48, 72 hpf (scale bar: 50 ?m). **(B, C)** Quantification of pH3+ ventricular CMs and mitotic index at 24, 48, and 72 hpf (n=5).

SUPPLEMENTARY FIGURES4 Knockdown of zebrafish Smarce1 leads to increased CM proliferation in developing zebrafish hearts. **(A)** Western Blot bands showing that Smarce1 protein is absent in smarce1 MO2 injected embryos at 96 hpf. **(B)** Confocal images of dissected hearts from control or smarce1 MO2 injected embryos with EdU incorporation at 96 hpf (scale bar: 50 ?m). **(C)** Confocal images of control or smarce1 MO2 morphants heart with pH3 staining displaying CM nucleus and proliferating CM at 96 hpf (scale bar: 50 ?m).
