# Supplementary material for: Smarce1 fine-tunes cardiomyocyte proliferation in the embryonic zebrafish heart
Source: Front Cell Dev Biol. 2025 Aug 29;13:1636944. doi: 10.3389/fcell.2025.1636944 (PMC12426013; doi:10.3389/fcell.2025.1636944)
Supplement: Supplementary file 1 [file Table1.docx]

**Supplementary Table S1.** List of materials

| **REAGENT or RESOURCE** | **SOURCE** | **IDENTIFIER** |
| --- | --- | --- |
| **Chemicals** | | |
| Proteinase K, recombinant, PCR Grade | Roche | Cat#3115844001 |
| 1-Phenyl-2-thiourea | Sigma-Aldrich | Cat#P7629 |
| Ethyl-3-aminobenzoate methanesulfonate salt | Sigma-Aldrich | Cat#A5040 |
| Paraformaldehyde (PFA) | Sigma-Aldrich | Cat#158127 |
| JB-4® Embedding Kit | Polyscience | Cat#00226-1 |
| Methyl Cellulose | Sigma-Aldrich | Cat#64632 |
| Trypsin (powder) | Biochrom | Cat#L2103 |
| VECTASHIELD® HardSet™ with DAPI | Vector Laboratories | Cat#H-1500 |
| Normal Goat Serum (NGS) | Sigma-Aldrich | Cat#S26-100ML |
| Triton^®^ X-100 | Serva Electrophoresis | Cat#37240 |
| Invitrogen^TM^ RNaseOUT™ Recombinant Ribonuclease Inhibitor | Thermo Fisher Scientific | Cat#10777-019 |
| Light Cycler 480 SYBR Green I Master Mix | Roche | Cyt#04887352001 |
| Nuclease free water | QIAGEN | Cat#129114 |
| cOmplete™, EDTA-free Protease Inhibitor | Roche | Cat#04693132001 |
| PhosSTOP™, Phosphatase Inhibitor | Roche | Cat#04906837001 |
| Protein Assay Dye Reagent Concentrate (Bradford) | Bio-Rad | Cat#5000006 |
| 8–16% Mini-PROTEAN® TGX™ Precast Protein Gels, 10-well | Bio-Rad | Cat#456-1104 |
| 10x Tris/Glycine/SDS | Bio-Rad | Cat# 1610732 |
| 10x Tris/Glycine Buffer | Bio-Rad | Cat# 1610734 |
| PageRuler™ Plus Prestained Protein Ladder | Thermo Fisher Scientific | Cat# 26619 |
| TWEEN 20 | Sigma-Aldrich | Cat#P1379 |
| Skim Milk Powder | Sigma-Aldrich | Cat#70166 |
| Amersham™ ECL Prime | Cytiva | Cat#RPN2232 |
| Immobilon-P PVDF-Membran | Millipore | Cat#IPVH00010 |
| Not I-HF | New England BioLabs | Cat#R3189L |
| **Antibodies** | | |
| Cyclin A1 | Aviva System Biology | Cat#OAAJ-01741 |
| Cyclin B1 | MybioSource | Cat#MBS9205589 |
| Cyclin E1 | Proteintech | Cat#11554-1-AP |
| p21_CDKN1A | Aviva System Biology | Cat#ARP30198_P050 |
| p27 Kip1 | GeneTex | Cat#GTX100446 |
| Anti-beta-actin | Sigma Aldrich | Cat#A5441 |
| Smarce1 | Aviva System Biology | Cat#ARP38224_P050 |
| Anti-phospho-Histone H3 (Ser10) | Millipore | Cat#06-570 |
| Anti-phospho-Histone H3 (Ser10) | Millipore | Cat#05-806 |
| Anti-mouse IgG, HRP-linked | Cell Signaling | Cat#7076S |
| Anti-rabbit IgG, HRP-linked | Cell Signaling | Cat#7074S |
| Goat anti-Mouse IgG1 Cross-Adsorbed Secondary Antibody, Alexa Fluor 488 | Thermo Fisher Scientific | Cat#A-21121 |
| Goat anti-Mouse IgM (Heavy chain) Cross-Adsorbed Secondary Antibody, Alexa Fluor 555 | Thermo Fisher Scientific | Cat#A-21426 |
| Goat anti-Rabbit IgG (H+L) Highly Cross-Adsorbed Secondary Antibody, Alexa Fluor 488 | Thermo Fisher Scientific | Cat#A-11034 |
| Goat anti-Rabbit IgG (H+L) Highly Cross-Adsorbed Secondary Antibody, Alexa Fluor 555 | Thermo Fisher Scientific | Cat#A-21429 |
| **Bacterial Strain** | | |
| One Shot TOP10 Electrocomp E.coli | Thermo Fisher Scientific | Cat#C404050 |
| **Critical Commercial Assays** | | |
| NucleoSpin RNA Plus XS | Machery-Nagel | Cat#MN740990250 |
| Click-iT® EdU Alexa Fluor® 488 Imaging Kit | Thermo Fisher Scientific | Cat#C10337 |
| Morpholino | GENE TOOLS, LLC | https://www.gene-tools.com |
| TOPO TA Cloning Kit Dual Promotor | Thermo Fisher Scientific | Cat#45-0640 |
| RNeasy Micro Kit | QIAGEN | Cat#74004 |
| Superscript III Reverse Transcriptase | Thermo Fisher Scientific | Cat#18080-085 |
| Q5^®^ High-Fidelity DNA Polymerase | New England BioLabs | Cat#M0491L |
| Isolate II PCR and Gel Kit | Biocat | Cat#BIO-52060 |
| Gateway Cloning | Thermo Fisher Scientific | Cat#11789100  Cat#11791020 |
| mMESSAGE mMASCHINE SP6 | Ambion | Cat#AM1340 |
| **Experimental Models: Animal** | | |
| Zebrafish | . | Tg(*myl7*:mcherry.*nls*) |
|  | . | Tg(*myl7*:GFP)) |
|  | Steffen Just lab (this paper) | Tg(*minUnc45b*:EGFP.CAAX) |
|  | . | Tg(*fli1*:GFP) |
|  | . | Tg(*myl7*:mcherry.CAAX) |
|  | Steffen Just lab (this paper) | Smarce1 TetON(*myl7*:AcGFP.nls) |
| **Oligonucleotides** | | |
| Oligo dTs | Eurofins | N/A |
| dNTP Mix | Thermo Fisher Scientific | Cat#R1122 |
| **Recombinant DNA** | | |
| pDonor221 | Thermo Fisher Scientific | Cat#12536017 |
| pCS2+ | Addgene | N/A |
| **Software and Algorithms** | | |
| GraphPad Prism (version 9) | GraphPad Software, La Jolla California, USA | https://www.graphpad.com:443 |
| ImageJ | NIH^42^ | https://imagej.nih.gov/ij/ |
| Imaris | Oxford Instruments | https://imaris.oxinst.com/ |

**Supplementary Table S2.** List of primer sequences

| **Experiment** | **Gene** | **Forward sequence (5´-3´)** | **Reverse sequence (5´-3´)** |
| --- | --- | --- | --- |
| Genotype | *smarce1* | TTCTGAGTGAGGTGGTGGTG | AGAGCCGTTTGAGCTCGTTA |
| Geteway cloning | *zsmarce1* | GGGGACAAGTTTGTACAAAAAAGCA GGCTTCGAAGGAGATAGAACCATGTCAAAGCGGCCCCCC | GGGGACCACTTTGTACAAGAAAGCTGGGTTTTATTATTGTGGCTGGGTGGGAG |
| Real-time PCR | *smarce1* | CCGAGCTGCTTCAAATCGAG | AGAGCCGTTTGAGCTCGTTA |
|  | *β-actin* | GCAGAAGGAGATCACATCCCTGGC | CATTGCCGTCACCTTCACCGTTC |
|  | *18s rRNA* | CACTTGTCCCTCTAAGAAGTTGCA | GGTTGATTCCGATAACGAACGA |
