# Supplementary figures and images for: Smarce1 fine-tunes cardiomyocyte proliferation in the embryonic zebrafish heart
Source: Front Cell Dev Biol. 2025 Aug 29;13:1636944. doi: 10.3389/fcell.2025.1636944 (PMC12426013; doi:10.3389/fcell.2025.1636944)

## Supplementary information

Figure. S1

A

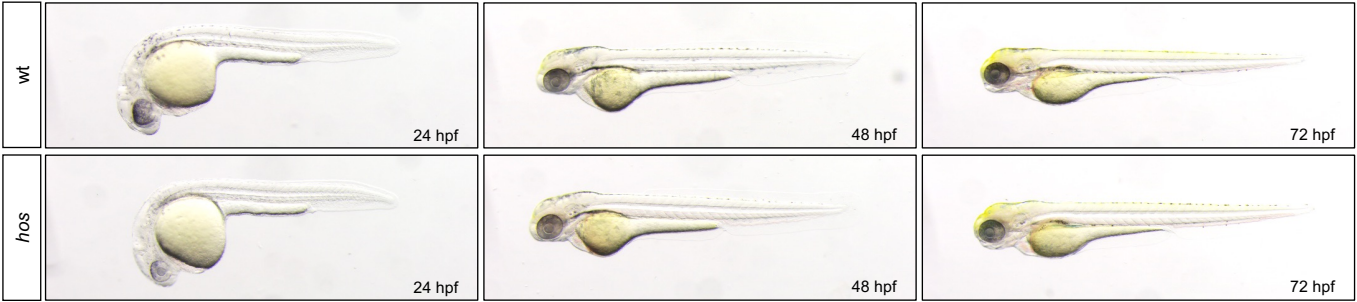

Figure. S2

A

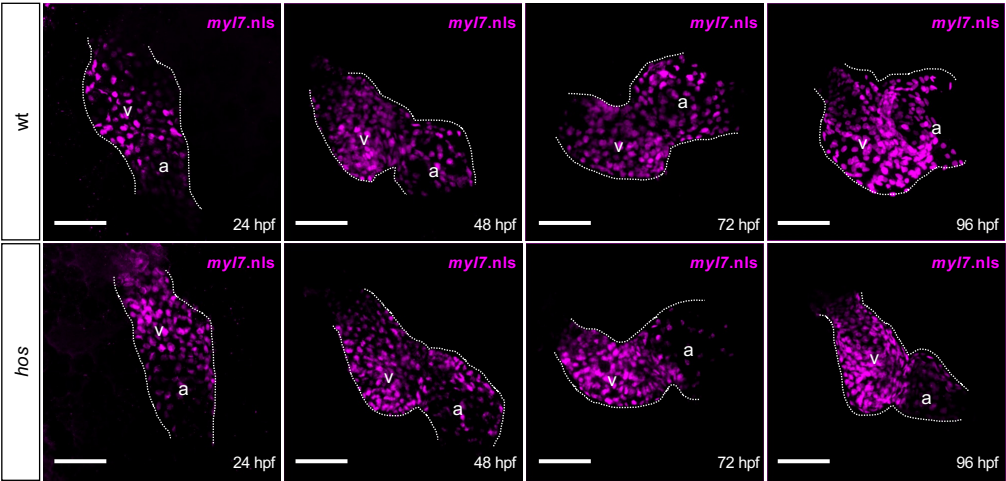

Figure. S3

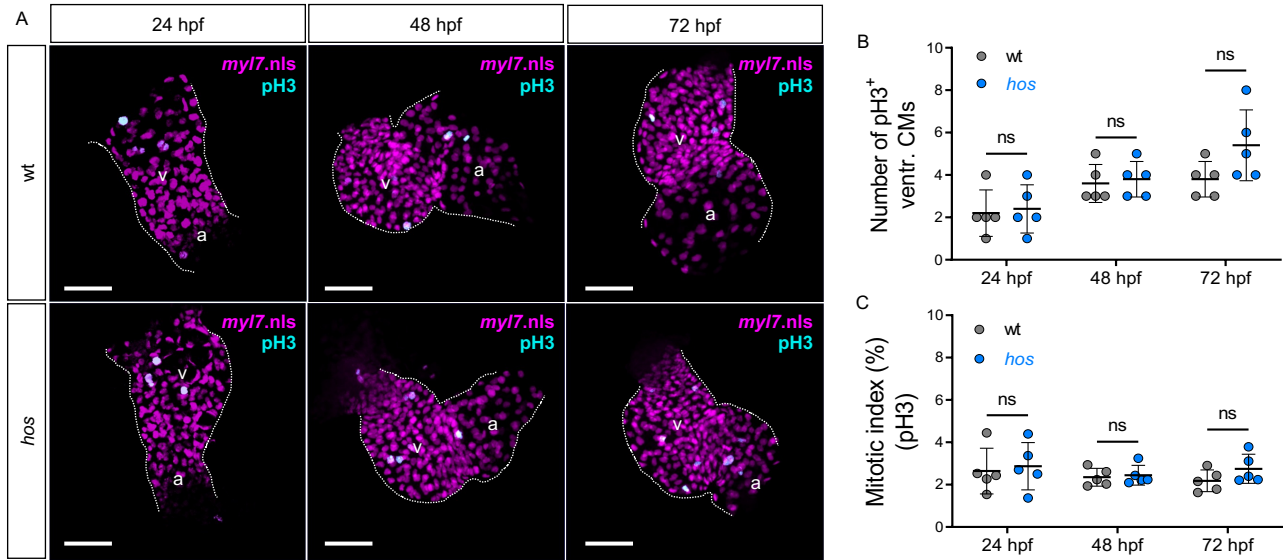

Figure. S4

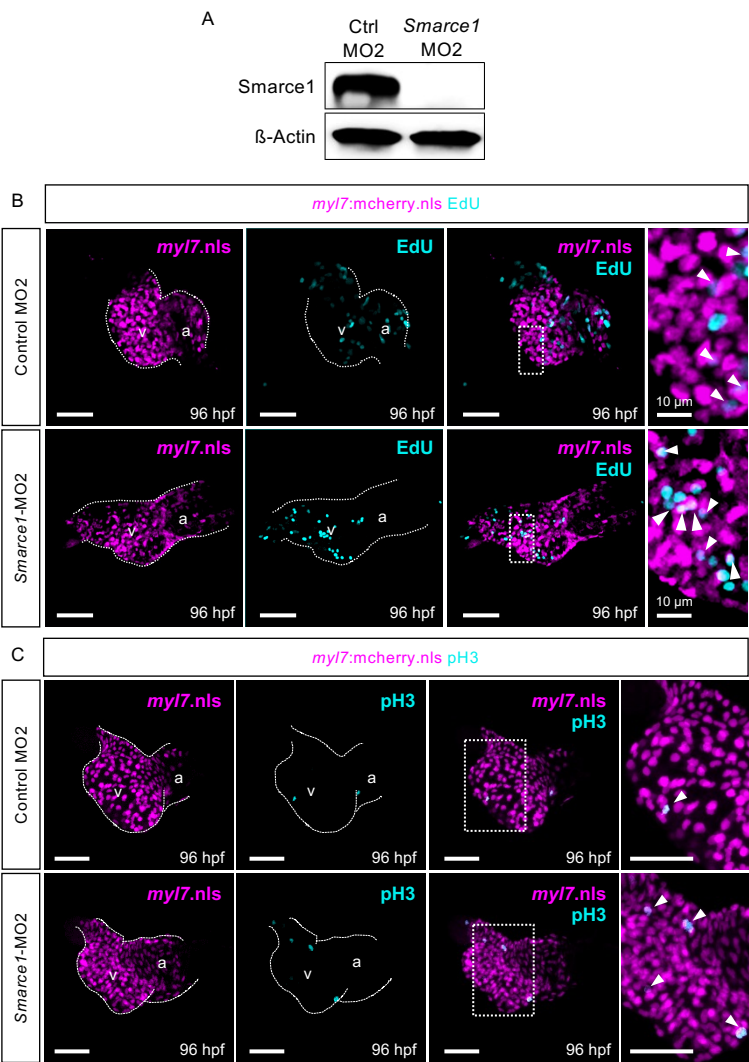

Supplement: Supplementary file 2 [file DataSheet1.pdf]
